# Artificial intelligence for early detection of diabetes mellitus complications via retinal imaging

**DOI:** 10.1007/s40200-025-01596-7

**Published:** 2025-04-12

**Authors:** Navid Sobhi, Yasin Sadeghi-Bazargani, Majid Mirzaei, Mirsaeed Abdollahi, Ali Jafarizadeh, Siamak Pedrammehr, Roohallah Alizadehsani, Ru-San Tan, Sheikh Mohammed Shariful Islam, U. Rajendra Acharya

**Affiliations:** 1 Nikookari Eye Center, Tabriz University of Medical Sciences, Tabriz, Iran; 2 Student Research Committee, Tabriz University of Medical Sciences, Tabriz, Iran; 3 Institute for Intelligent Systems Research and Innovation (IISRI), Deakin University, 75 Pigdons Rd, Waurn Ponds, VIC 3216 Australia; 4 Faculty of Design, Tabriz Islamic Art University, Tabriz, Iran; 5 National Heart Centre Singapore, Singapore, Singapore; 6 Duke-NUS Medical School, Singapore, Singapore; 7 Institute for Physical Activity and Nutrition, School of Exercise and Nutrition Sciences, Deakin University, Melbourne, VIC Australia; 8 Cardiovascular Division, The George Institute for Global Health, Newtown, Australia; 9 Sydney Medical School, University of Sydney, Camperdown, Australia; 10 School of Mathematics, Physics, and Computing, University of Southern Queensland, Springfield, QLD 4300 Australia; 11 Centre for Health Research, University of Southern Queensland, Springfield, Australia

**Keywords:** Diabetes mellitus, Artificial intelligence, Diabetic retinopathy, Diabetes complications, Retina

## Abstract

**Background:**

Diabetes mellitus (DM) increases the risk of vascular complications, and retinal vasculature imaging serves as a valuable indicator of both microvascular and macrovascular health. Moreover, artificial intelligence (AI)-enabled systems developed for high-throughput detection of diabetic retinopathy (DR) using digitized retinal images have become clinically adopted. This study reviews AI applications using retinal images for DM-related complications, highlighting advancements beyond DR screening, diagnosis, and prognosis, and addresses implementation challenges, such as ethics, data privacy, equitable access, and explainability.

**Methods:**

We conducted a thorough literature search across several databases, including PubMed, Scopus, and Web of Science, focusing on studies involving diabetes, the retina, and artificial intelligence. We reviewed the original research based on their methodology, AI algorithms, data processing techniques, and validation procedures to ensure a detailed analysis of AI applications in diabetic retinal imaging.

**Results:**

Retinal images can be used to diagnose DM complications including DR, neuropathy, nephropathy, and atherosclerotic cardiovascular disease, as well as to predict the risk of cardiovascular events. Beyond DR screening, AI integration also offers significant potential to address the challenges in the comprehensive care of patients with DM.

**Conclusion:**

With the ability to evaluate the patient’s health status in relation to DM complications as well as risk prognostication of future cardiovascular complications, AI-assisted retinal image analysis has the potential to become a central tool for modern personalized medicine in patients with DM.

## Introduction

Diabetes mellitus (DM) affects more than 422 million people globally and is among the top ten causes of mortality and morbidity [1, 2]. It significantly increases the risk of macrovascular complications, including atherosclerotic cardiovascular diseases such as coronary heart disease, cerebrovascular disease, and peripheral arterial disease (PAD), with mortality rates from cardiovascular diseases being two to six times higher in individuals with diabetes than in non-diabetic populations [1] Additionally, it predisposes patients to microvascular complications, such as peripheral neuropathy [3], nephropathy, where the prevalence of end-stage kidney disease is ten times greater in diabetic populations [4], and retinopathy.

Diabetic retinopathy (DR) is the leading cause of preventable adult blindness [5] with a prevalence of 34.6% among diabetic patients [6]. DR can be categorized into non-proliferative DR (NPDR) and proliferative DR (PDR). NPDR is the early stage of DR, in which existing retinal blood vessels weaken, leading to microaneurysms and possible fluid leakage. It progresses from mild to severe stages without new blood vessel formation. However, PDR refers to the advanced stage of DR characterized by the growth of new abnormal blood vessels on the retina or optic disc, which can lead to serious vision complications [7]. The incidence of sight-threatening diabetic macular edema (DME), where fluid accumulates in the macula due to leaking retinal blood vessels, causing swelling and potential vision loss [8, 9], can occur at any stage and increases as DR progresses [5]. With the rising prevalence and incidence of DM, the burden of DR and the consequent vision loss has increased. Globally, 1.4 million patients with DM have severe NPDR and PDR and will require treatment to prevent or slow further ‎vision deterioration [6]. Therefore, the term referable DR refers to cases in which DR has progressed to a level requiring specialist referral, typically encompassing moderate NPDR or worse, or the presence of DME [10]. Preemptive systematic and continual screening of asymptomatic diabetic patients, with early initiation of antiproliferative treatment where indicated, is obligatory for preventing clinical sequelae and reducing the human costs of DR [11].

Retinal vasculature is the only part of the human body’s microcirculation and can be visualized noninvasively [12]. The retina is a veritable “window” to the state of health of the microcirculation, providing insights into DR as well as other diabetic macrovascular and microvascular complications such as neuropathy and nephropathy [13]. Fundoscopic retinal examination is traditionally used in clinics to screen for DR. However, the introduction of digital fundus photography, which enables offline analysis of retinal images by experts, has contributed to the successful implementation of national DR screening programs. With the advent of artificial intelligence (AI) applications in medicine, especially in the interpretation of digital medical images (which are by nature objective and accessible), many AI-based systems utilizing AI’s ability to handle high-throughput, ‎complex image data have been developed for the detection of DR using digitized retinal images and are now clinically adopted [14, 15].

**Fig. 1:**
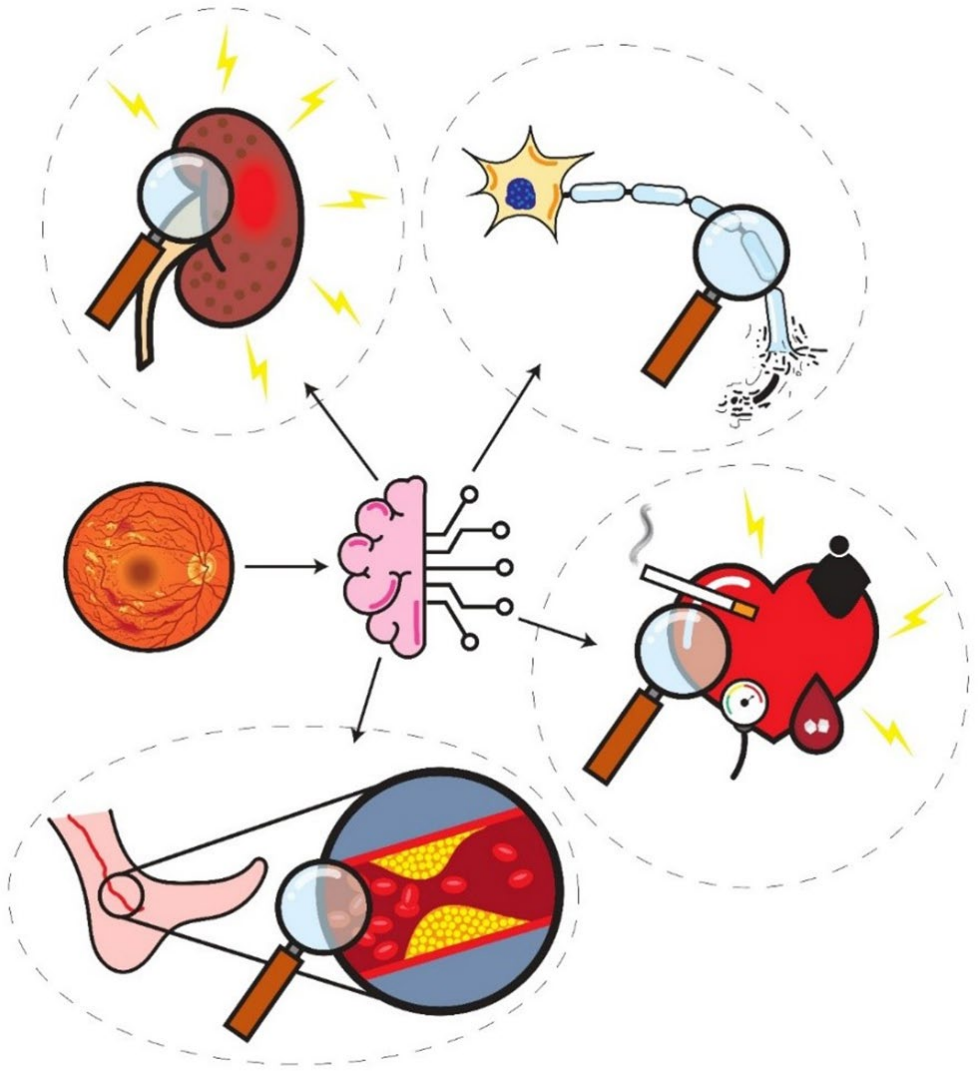
Illustration of various diabetic complications diagnosed by retinal imaging using AI. (Graphical abstract)

Beyond DR screening, AI integration also holds immense potential for addressing the challenges associated with the comprehensive care of patients with DM complications. As mentioned above, retinal vasculature images contain diagnostic and prognostic clues to the microvascular and macrovascular health of the whole body. In contrast to existing review studies centered solely on DR within the context of DM, the retina, and AI, our study takes a broader approach [16–24]. Our review takes a comprehensive perspective, covering all DM-related complications detectable through retinal imaging rather than focusing solely on DR, as in most existing studies. We explored AI’s potential to identify a broad range of complications, including DME as well as cardiovascular, neurological, and kidney diseases. Additionally, most studies emphasize diagnostic capabilities, with little attention given to the role of AI in predicting disease progression or guiding personalized treatments. Real-world implementation challenges, including adapting AI models to diverse healthcare settings, data privacy concerns, and regulatory hurdles, are underexplored. Ethical issues, such as algorithm bias and equitable access to AI tools, have received minimal discussion, especially in the context of low-resource settings where DM complications are prevalent. This narrative review aims to address these gaps by exploring broader applications of AI in diabetes care, synthesizing insights into multi-complication detection, and highlighting barriers to clinical adoption. Various DM-related complications are visualized in Fig. 1.

## Literature search method

We performed our search in PubMed, Scopus, and Web of Sciences databases for scholarly articles published in the English language until June 2024, using various combinations (by using Boolean operators: AND, OR, NOT) of the following main keywords: “diabetes mellitus,” “retina,” “artificial intelligence.” Other keywords were “diabetic care,” “diabetes complications,” “diabetic foot,” “diabetic nephropathy,” “diabetic neuropathy,” “diabetic retinopathy,” “diabetic macular edema,” “cardiovascular risk assessment,” “peripheral arterial disease,” “fundus image,” “personalized medicine,” “deep learning,” “machine learning,” and “machine vision”.

In selecting articles for inclusion in our study, we employed an approach focused on identifying peer-reviewed original studies written in English with clear research goals, meticulous methodology, and extensive technical details. In our evaluation process, we defined the ‘technical details’ to include the technological aspects and analytical methods used in the studies. We also excluded non-peer-reviewed and low-quality articles with unclear research goals, duplicates, editorial articles, and conference abstracts. This entailed examining the types of AI algorithms deployed, data-processing techniques, specific retinal imaging methods, and any advanced computational strategies implemented. We also discuss software applications, statistical techniques, and validation procedures in these studies. Our focus on these technical details is to ensure a comprehensive assessment of the methodologies and technological advancements in AI applications in diabetic retinal image analysis.

## Diabetic retinopathy and diabetic macular edema

### DR screening

In a landmark study by Abramoff et al., an AI system based on supervised machine learning (ML) with logistic regression attained 96.8% sensitivity, 59.4% specificity, and 0.937 (95% CI, 0.916–0.959) AUC (a generic term referring to the area under a curve, typically used to evaluate classifier performance) for the detection of referable DR. Despite its modest specificity, the system was projected to halve the screening burden compared with manual screening by experts [10]. The team developed a deep learning (DL) model, based on convolutional neural network (CNN) architectures inspired by AlexNet and VGGNet, to enhance the detection of referable DR. This model demonstrated a specificity of 87% (95% CI: 84.2–89.4%) and an AUC of 0.980 (95% CI: 0.968–0.992), achieving these improvements without compromising sensitivity [25]. EyeArt and Retmarker, two AI systems designed to detect referable DR, reported sensitivities of 93.8% (92.9-94.6%) and 85% (83.6-86.2%), respectively, and have been linked to reduced costs in DR screening [26]. Other economic models studies also have also shown that semi-automated and fully automated screening methods by human experts are more cost-effective than traditional manual screening [27, 28]. IDx-DR, the first medical device implementing AI for detecting moderate and severe DR, was approved by the FDA in 2018 [29]. A cost-effectiveness analysis of a nationwide DR screening program in China involving 251,535 participants revealed that AI models need a sensitivity of at least 88.2% and specificity of 80.4% to be cost-effective. The most cost-effective scenario showed a higher sensitivity (96.3%) but lower specificity (80.4%) than the most accurate model (93.3% sensitivity and 87.7% specificity). This study showed that urban regions and younger populations demand higher sensitivity for optimal cost-effectiveness [30]. Additionally, another cost-effectiveness analysis revealed that implementing AI-based DR screening in primary care for both non-indigenous and Indigenous Australians with DM is highly effective and cost-saving, with significant reductions in blindness cases and healthcare costs projected over 40 years [31].

Recent studies have highlighted the potential of AI to improve DR screening and diagnosis. In a study by Riazi-Esfahani et al. [32], 84 eyes of 57 individuals were analyzed to differentiate between normal eyes, NPDR, and PDR. They utilized optical coherence tomography (OCT) images, evaluated an ML-based segmentation system, and found significant differences in the retinal layer area and smoothness index (SI) across the groups. Specifically, the inner nuclear layer (INL) area was the most effective in distinguishing DR stages, with an accuracy of 87.6%, while the SI of the inner plexiform layer (IPL) in the nasal zone distinguished PDR from NPDR with 97.2% accuracy. The DL model developed by Saranya et al. [33] for detecting DR showed remarkable metrics. The model’s accuracy, sensitivity, specificity, and F1-scores (The harmonic mean of precision and recall used to evaluate the balance between false positives and false negatives in a binary classification) were 97.54%, 90.34%, 98.24%, and 93.28% on the MESSIDOR dataset, and 96.32%, 95.73%, 97.12%, and 96.74% on the E-ophtha Ex dataset. In another study, an AI screening model for referable DR in Rwanda demonstrated 92% (95% CI, 86.3–96.8%) sensitivity, 85% (95% CI, 75.1–88.2%) specificity, and 33.2% of patients were referred for follow-up [34]. Similarly, in Tanzania, a trial involving 2364 participants aims to improve the follow-up rates for referable DR cases using AI-supported screening pathways [35]. Another study comparing 21 AI algorithms for DR screening found varying performances. The mean agreement for referable DR was 79.4%, with sensitivity and specificity of 77.5% and 80.6%, respectively. These findings emphasize the need for real-world validation before clinical application [36]. Additionally, an AI model developed in Greenland for DR screening showed an AUC of 0.99 but requires further optimization for practical clinical application [37]. Lastly, AI analysis of retinal microvascular changes demonstrated the potential for the early diagnosis of diabetic complications by identifying significant retinal abnormalities [38].

In low-income countries with limited healthcare resources, the lack of established DM screening programs and insufficient blood glucose control results in a higher incidence of DM-related complications. In one study conducted in sub-Saharan Africa, the rate of progression from no DR to sight-threatening DR (STDR) was five times higher than in Europe [39, 40]. However, universal annual DR screening for all patients with DM remains elusive goal [40]. The solution may lie in using technological advancements, such as AI-based analysis of digital fundus photographs, which has the potential to reduce the costs of nationwide screening programs while delivering comparable or even superior diagnostic performance [6, 41, 42]. However, whether models developed and validated in countries with different ethnic compositions can be effectively applied to local populations is still a valid concern. In a study by Bellemo et al. [43], an ensemble model combining adapted VGGNet and ResNet architectures trained on a Singaporean database of color retinal images, demonstrated robust diagnostic performance in a real-world diabetic population in Zambia. Specifically, the model achieved an AUC of 0.973 (95% CI: 0.969–0.978) for detecting referable DR, with a sensitivity of 99.42% (95% CI: 99.15–99.68) for STDR and 97.19% (95% CI: 96.61–97.77) for DME. Separately, a real-world prospective interventional cohort study in Thailand utilized a DL-based system to detect STDR and reported an accuracy of 94.7% (95% CI 93.0–96.2), compared to 93.5% (91.7–95.0) achieved by human experts [44]. Moreover, among novel cost-effective approaches, retinal images can now be captured using smartphone-based retinal cameras. In one study, this method proved feasible when using the EyeArt software, an ensemble of deep artificial neural networks, which achieved a sensitivity of 99.1% (95% CI: 95.1–99.9) and a specificity of 80.4% (95% CI: 73.9–85.9) for detecting STDR based on online-processed smartphone retinal images evaluated against human expert assessments [45].

### Advanced DR screening for DME

DME, an accumulation of fluid in the macula, can lead to visual loss at any stage of DR and mandates referral to specialists. Definitive diagnosis requires macular thickness measurement using OCT or visualization of edema on fluorescein angiography, which is the gold standard diagnostic tool. Hard exudates within one optic disc diameter on the color fundus photograph (CFP) are a surrogate for DME [46], with a significant false positive rate of up to 42% in the UK screening program [47]. The Danish guidelines recommend OCT as a second-line screening method for DR [48]. Diagnostic criteria for DME, as identified through OCT imaging, include central retinal thickness (CRT) > 250–300 μm, presence of intraretinal or subretinal fluid, hyperreflective dots, and disruption of retinal layers [49]. Moreover, diagnostic criteria for DME, as identified through fluorescein angiography, include leakage of fluorescein dye, areas of capillary nonperfusion, and evidence of macular ischemia [50].

AI plays a significant role in diagnosing and classifying DME [23]. A DL model trained on fundus photographs attained an AUC of 0.89 (95% CI: 0.87–0.91) for discriminating center-involved DME, with superior specificity and positive predictive value compared to human experts [51]. More DL systems have been developed for the automatic detection of DME on OCT images, which can also discern grades of severity, ranging from diffuse retinal thickening cystoid macular edema to serous retinal detachment [52]. A study by Manikandan et al. reported 96% (95%CI: 0.94–0.98) and 94% (95% CI: 0.90–0.96) sensitivities for AI-assisted DME detection based on OCT and fundus images, respectively [53]. Similarly, another study by Lam et al. evaluated the performance of various DL models in detecting DME using fundus photography and OCT images. They reported a pooled area under the receiver operating characteristic curve (AUROCs) of 0.964 (95%CI: 0.964–0.964) for fundus photography-based algorithms and 0.985 (95%CI: 0.985–0.985) for OCT-based algorithms, with sensitivities of 92.6% and 95.9% and specificities of 91.1% and 97.9% [54]. AUROC measures the ability of a classifier to distinguish between classes across all thresholds, with values closer to 1 indicating better performance. Another investigation reported a pooled 96.0% (95% CI: 93.9–97.3%) sensitivity and 99.3% (95% CI: 98.2–99.7%) specificity for DME detection using DL with OCT images [55]. In a comparison with the other two DL models, the Optic-Net model (98% accuracy, 100% specificity) outperformed Dense-Net (94% accuracy, 96% specificity) for OCT-based DME classification [56]. Liu et al. [57] trained a faster R-CNN model with ResNet101 backbone on more than 50,000 labeled fundus images and 20,000 OCT B-scans acquired from patients across multiple centers and reported a remarkable 97.78% sensitivity, 98.38% specificity, and 0.981 (95%CI: 0.966–0.990) AUC for detection of referable DR; and 91.30% sensitivity, 97.46% specificity, and 0.944 (95% CI 0.922–0.962) AUC for detection of DME. The incremental diagnostic utility of OCT-based AI analysis―combined CFP plus OCT screening-detected cases of DME that would have been missed on CFP analysis alone― supports the addition of OCT to standard CFP-based AI screening programs; however, cost-effectiveness should be further assessed.

AI evaluation of OCT angiography (OCTA), which generates angiographic images of retinal vessels from motion imaging of volumetric blood flow signals without contrast, has recently attracted attention. Ryu et al. [58] compared two models for detecting early DR using OCTA. The first model was a ML-based classifier that combined segmentation—via a U-Net capable of isolating blood vessels and the foveal avascular zone in OCTA images—followed by feature extraction and classification. The second model employed a CNN-based classifier that processed the OCTA images directly using a ResNet101 architecture. Notably, the CNN-based model achieved excellent performance, with an accuracy of 91–98%, sensitivity of 86–97%, specificity of 94–99%, and AUC values between 0.919 and 0.976, comparing favorably against ultra-widefield fluorescein angiography.

### Grading of DR severity

**Fig. 2:**
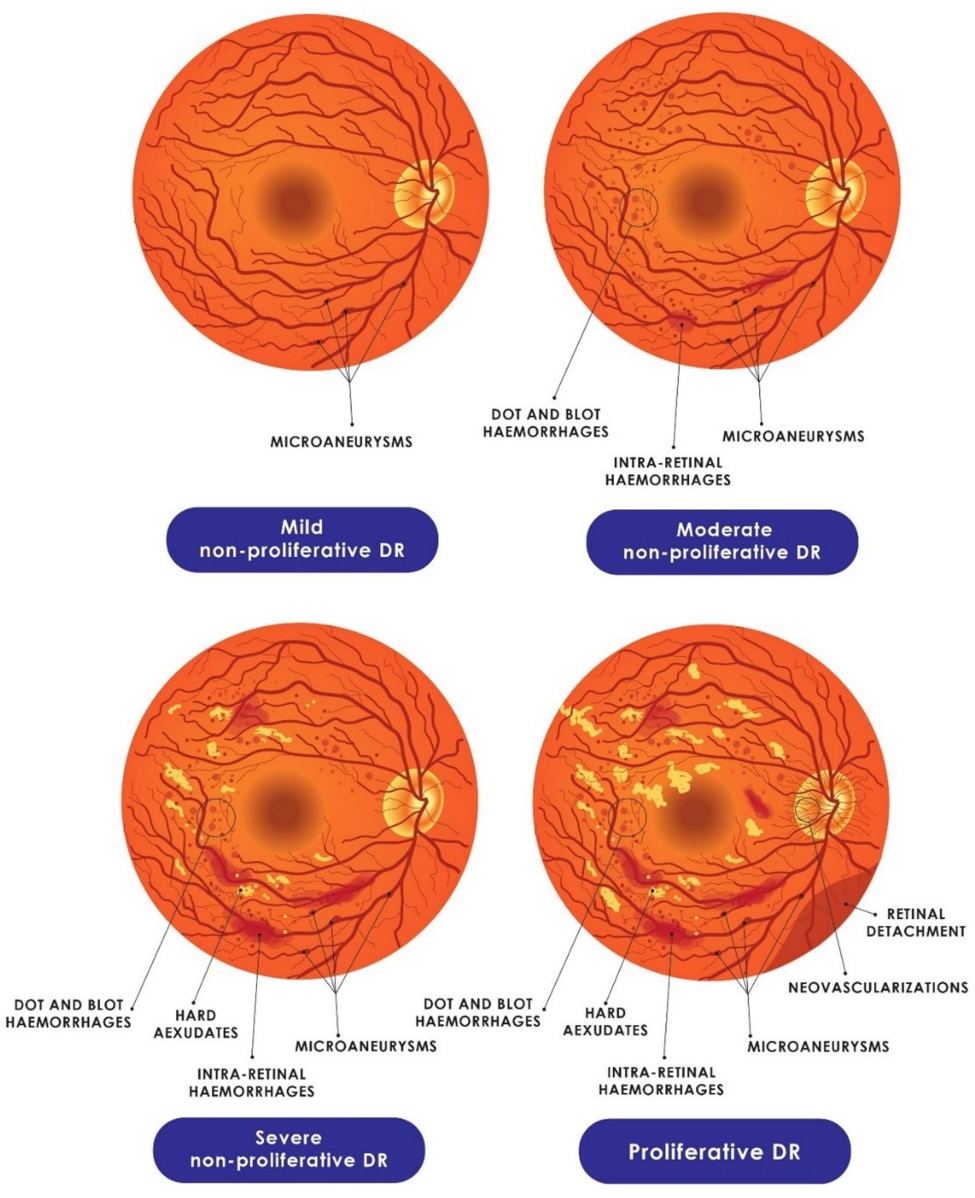
Schematic representation of fundoscopy showing the DR grading

Classification of CFP images into distinct DR severity grades ―no retinopathy, mild NPDR, moderate NPDR, severe NPDR, and PDR [24]― based on retinal vascular changes can provide valuable insights into disease progression and prognosis [59]. This is traditionally performed through expert manual fundus examinations for DR [60], a resource that is both limited and not easily accessible. Promising AI-based tools can categorize DR grades and reduce healthcare costs and burdens [27, 61]. Gulshan et al. [62] developed a CNN model with Inception-v3 architecture and transfer learning (TL) which outputs five independent binary classifiers for DR grading. The model attained good performance for grading DR, and achieved 84.0% (95% CI, 75.3-90.6%) sensitivity and 98.8% (95% CI, 98.5-99.0%) specificity for detecting severe or worse DR. Additionally, it attained 90.8% (95% CI, 86.1-94.3%) sensitivity and 98.7% (95% CI, 98.4- 99.0%) specificity for detecting DME. Takahashi et al. [63] trained a modified GoogLeNet model on 9,939 fundus photographs (four 45° color fundus photographs per eye) using a refined Davis grading protocol. When tested on 496 images (5% of the dataset), the model achieved 81% accuracy (correctly classifying 402 images) and a prevalence- and bias-adjusted Fleiss’ kappa (PABAK) of 0.64 compared with the modified Davis grading. Against a real prognosis grading, the model attained a PABAK of 0.37 and 96% accuracy. Other researchers have also reported high sensitivity and specificity rates for DR grading using various models [64–66]. Figure 2 shows a schematic illustration of various DR severity grades.

In fundus examination, DR–induced vascular changes often manifest heterogeneously across the retina. Nevertheless, traditional manual analyses generally classify the entire fundus image according to the most severe lesion identified in any region of the image. With AI analysis of CFP images, the basis for severity grading is not apparent, which limits the explainability of AI decision-making. To address this, researchers have developed DL neural networks for lesion-based classification [62, 66, 67]. Wang et al. [68] trained a CNN-based DR lesion classifier, Lesion-Net, on 12,252 fundus images of patients with DM. The model contains two branches: the inferior branch uses a fully convolutional network (FCN-32s) for lesion segmentation predictions of eight types of common DR lesions to diagnose and stage DR, and the superior branch integrates convolutional layers from the Inception-v3 architecture to classify referable vs. non-referable DR classes. Lesion-Net attained a good AUC of 0.943, sensitivity of 90.6%, and specificity of 80.7% for the five-stage DR grading as well as an improved AUC of 0.936 vs. 0.928 for the internal test set and 0.977 vs. 0.964 for the external test set for binary referable vs. non-referable DR classification. According to a recent study, the Inception-v3 model, trained on a dataset of 35,126 retinal images, delivered the best performance for DR diagnosis among ML and DL models, yielding an accuracy rate of approximately 97% in feature extraction [69]. Concurrently, the MobileNetV3 model has been identified as optimal for data classification tasks in the same domain, achieving an accuracy of 98.56% [70].

Sandhu et al. combined OCT and OCTA images with primary clinical and demographic data collected from 111 patients to train an AI model for DR screening and staging [71]. They developed a novel computer-aided design system to grade NPDR into mild and moderate stages. They reported 98.7% accuracy, 100% sensitivity, 97.8% specificity, 99% differential scanning calorimetry (DSC), and 0.981 AUC (progressive improvements in almost all metrics were observed as OCTA, clinical, and demographic data were incrementally added to the model). Wang et al. [72] used ultra-widefield fluorescein angiographic images from 399 patients to train an AI model for differentiating normal retina, NPDR, and PDR. The resulting developed model attained a classification accuracy of 88.50%.

### Individualized DR screening

The current guideline-based practice for DR screening involves annual fundoscopic examination. However, this practice fails to account for the heterogeneity of the clinical course of different patients. For example, only 11% of individuals with mild NPDR in both eyes progress to STDR each year, whereas all patients should undergo annual screening [73]. Given the increasing incidence of DM and projected increase in DM-related healthcare resource utilization, individualized screening at varying intervals and approaches (home visit, AI-assisted, or specialized screening) may be more cost-effective as an alternative to routine annual DR screening.

Mathematical models have been developed to predict the risk of progression from non-DR or mild DR to STDR. Aspelund et al. used six parameters―sex, DM type, duration of DM, glycated hemoglobin, blood pressure, and the existence and grading of DR―to statistically model optimal DR screening intervals [74–76]. Compared to routine annual DR screening, their model, along with those developed by other researchers, achieved a 40–60% reduction in the number of screening visits, which resulted in an 11–40% increase in the incidence of STDR detected at subsequent screening visits [74–76]. Moreover, AI can potentially improve the prediction accuracy of mathematical models using the least amount of data. Piri et al. analyzed demographic, laboratory, and comorbidity data of more than 1.4 million patients with DM using supervised ML models and artificial neural networks where only a routine blood test could predict DR with an accuracy of 92.76% [76]. These observations suggest that risk stratification without the need for retinal imaging could facilitate the early diagnosis of DR while reducing the need for mass screening, notably in regions with poor infrastructure and low compliance.

### Individualized DR follow-up

There is considerable variability in the progression of DR between patients with DM, even among those diagnosed with the same DR grade. A clear understanding of the anticipated progression of the disease greatly enhances the ability to develop the most effective screening and treatment plans for each patient. This enables customization of treatment options, scheduling of visit frequencies, and timing of interventions to optimize individual patient outcomes [77]. Skevofilakas et al. [78] developed a decision support system (DSS) for assessing DR progression using clinical data collected from the electronic health records of 55 type I DM (T1DM) patients who were followed over five years. The DSS used strongly linked risk factors―age, DM duration, glycated hemoglobin, cholesterol, triglyceride, hypertension (HTN), incidence rate, and DM treatment duration―to predict DR progression. Receiver operating characteristic (ROC) analysis showed that the most accurate primary classifier had an accuracy of 97%, while the overall combined DSS had higher sensitivity, specificity, and accuracy.

Developing an AI model based on images, demographics, and clinical information to predict the risk of progression enables clinicians to make informed decisions. Arcadu et al. [79] developed an algorithm to predict the worsening of DR using 7-field CFPs acquired from patients with DM who were monitored over two years. The baseline CFPs were used to train the model to indicate the presence of 2-step or greater worsening of DR on the Early Treatment DR Scale during the follow-up visits. Based on an Inception-v3 architecture, separate deep CNNs (DCNN) were trained in parallel for each of the seven fields using TL initialized with ImageNet weights. Ultimately, the generated probabilities were consolidated using random forests (RF) to enhance the overall prediction accuracy. The trained DL model predicted worsening of DR at multiple intervals. It achieved an AUC of 0.68 ± 0.13, sensitivity of 66% ± 23%, and specificity of 77% ± 12% at 6 months. At 12 months, it achieved an AUC of 0.79 ± 0.05, sensitivity of 91% ± 8%, and specificity of 65% ± 12%. For the 24-month prediction, the model achieved an AUC of 0.77 ± 0.04, a sensitivity of 79% ± 12%, and a specificity of 72% ± 14%. Their results provided evidence for the use of AI in refining and personalizing the prognostication of DR. The ability of the AI model to accurately predict disease progression, even among individuals within the same DR grade as assessed by traditional manual methods, shows its potential to enhance clinical decision-making and patient management. Notably, the aggregate result was also superior to the output of each field-specific DCNN. Furthermore, they discovered that the peripheral parts of the retina contributed more to the predictions than the central regions, with the model performance dropping significantly when the peripheral zones were excluded. Additionally, the attribution map showed that the model could differentiate between microaneurysms, hemorrhages, and hard exudates, providing a foundation for potential lesion-specific analyses and predictions. Finally, the results of this study suggest that, in contrast to traditional manual grading assessments, which can only predict disease outcomes in a group of patients with similar signs and symptoms, DL algorithms can approximately predict the course of DR in a single patient.

Similarly, another study improved the automated ML models to predict DR progression from retinal images. The models were trained and validated on images with mild or moderate NPDR over three years using images from a tertiary diabetes center. The models achieved an Area Under the Precision-Recall Curve (AUPRC), which summarizes the performance of a classifier in handling imbalanced datasets by focusing on precision and recall trade-offs across thresholds of 0.717 for mild NPDR and 0.863 for moderate NPDR, with sensitivity and specificity ranging from 0.63 to 0.80 [80]. Moreover, another trial evaluated the effectiveness of autonomous AI-based diabetic eye screening at the point-of-care in increasing examination completion rates among youth with type 1 and type 2 diabetes mellitus. This study compared AI-assisted examinations with traditional eye care provider referrals and found that the completion rate was significantly higher in the AI group (100%) than in the control group (22%). This study showed that AI could significantly improve DR follow-up adherence in a diverse youth population [81].

### DR treatment: who to treat and how to treat?

Intravitreal injection of anti-vascular endothelial growth factor (VEGF) medications, such as ranibizumab, bevacizumab, and aflibercept, is an indicated treatment for STDR, especially DME [82–85]. OCT is often used to monitor therapeutic responses. Through the analysis of OCT images, AI models can predict individual patient responses to anti-VEGF therapy and potentially facilitate personalized treatment approaches for DME [86]. Advanced AI algorithms have been developed to evaluate OCT parameters―central macular fluid volume, integrity of the ellipsoid zone, intraretinal fluid, subretinal fluid, hyperreflective retina foci, and external limiting membrane [87]―to predict visual acuity trajectories in DME, thereby providing clinicians with objective parameters for DME diagnosis and follow-up. Liu et al. [88] developed an ensemble ML system that consisted of DL models and five classical ML (CML). They trained AlexNet, VGG16, ResNet18, and an ensemble of three DL architectures on a dataset of 304 pretreatment OCT images of patients with DME. Fifteen OCT features generated by the DL ensemble model were then used to train conventional ML algorithms―Least Absolute Shrinkage and Selection Operator (LASSO), support vector machine (SVM), decision tree (DT), and RF― to predict post-treatment central foveal thickness (CFT) and best-corrected visual acuity (BCVA) values one month after three months of anti-VEGF injections [88]. However, the model failed to accurately predict post-treatment CFT and BCVA values, suggesting that OCT images alone were insufficient as the sole model inputs. However, adding further clinical information associated with treatment outcomes in CML models improves prediction accuracy.

An AI system’s prediction of a poor treatment response may justify the adoption of alternative therapeutic strategies, thereby minimizing unnecessary risks and costs, while ensuring that patients who could benefit from appropriate treatments are not excluded. In a retrospective study, Gallardo et al. [89] developed an ML system to assess the burden of anti-VEGF treatment―defined as low, moderate, and high based on the interval of injections―in a treat-and-extend (T&E) regimen for DME and retinal vein occlusion using demographic data and OCT images obtained from patients at two consecutive clinic visits [89]. The proposed RF-based supervised ML model predicts the 1-year treatment requirement with a reasonable AUC so that the decision-making process is rendered interpretable. In particular, all features related to intraretinal fluid are important for predicting low and high treatment demands.

In addition to predicting treatment response, AI can be applied to the direct planning of therapeutic procedures. Focal or grid laser photocoagulation, in which a series of controlled photocoagulations are delivered to the pathological areas of the retina [90, 91], is another indicated treatment for DME and PDR. This treatment induces regression of neovascularization by normalizing oxygen partial pressures in the peripheral avascular regions of the retina. As a result, the rates of vitreous hemorrhage and membrane formation were reduced.

Treatment efficacy is highly dependent on the siting and dosing of the administered photocoagulates [92]. Standard predetermined patterns for photocoagulation cannot account for individual differences in the shapes and patterns of macular edema and anatomical variations of the retinal vasculature [90, 93]. Furthermore, manual mapping of the coagulation pattern requires surgical expertise and considerable time [93]. AI can be used to automate retinal segmentation, such that only personalized, predetermined areas of the retina are coagulated, thereby increasing the precision of laser photocoagulation and minimizing unwanted side effects. Novel AI software has generated personalized, high-quality coagulation maps by processing patient information. The system enhances precision in localizing the exact burn points and controlling the power delivered compared to manual methods, resulting in a nine-fold reduction in laser burns beyond the edema borders, shortened procedural preparation time, and fewer postoperative complications [93].

## Beyond the eye: systemic microvascular and macrovascular complications

Table 1 summarizes studies on AI models developed using retinal images to detect DM complications or sequelae outside the eye. The explanations are detailed in the following sections.

### Predicting cardiovascular risk using retinal photographs

DM increases the risk of cardiovascular events by two to four folds [1, 94], and cardiovascular diseases remain the leading cause of death in individuals with DM. Evaluating future cardiovascular risks may help guide treatment decisions and enable timely preventive measures. In response to this need, risk calculators have been developed using traditional statistical models that incorporate both clinical factors (e.g., age, sex, body mass index, and blood pressure) and laboratory measures (e.g., eGFR, HbA1c, HDL, LDL, and triglycerides) to generate predictions. Notably, such models are not specific to the DM population. Based on the same information, an AI model modestly improved risk prediction performance, increasing the AUC from 0.69 to 0.75 [95].

Retinal parameters have been shown to correlate with cardiovascular outcomes In DM patients, independent of underlying risk factors [96]. The abundance of retinal image databases from patients with DR has enabled researchers to extract new associations using AI. For instance, Poplin et al. trained an Inception-v3 model on 284,335 retinal images and reported 0.70 AUC for image-based prediction of major cardiovascular events [97]. Moreover, the accuracy of cardiovascular risk prediction can be enhanced using combined retinal images and accessible clinical and demographic data [98]. Finally, cardiovascular risk assessment could be part of DR screening in the path toward holistic diabetes care.

### Beyond the optic nerve: predicting diabetic neuropathy

Similar to DR, diabetic neuropathy is a common microvascular complication of DM. It causes skin ulcers, increases the risk of limb amputation, and reduces quality of life, accounting for 27% of annual DM-related healthcare costs [99–101]. The gold standard for diagnosing diabetic neuropathy is a combination of clinical examinations such as the monofilament test and electrophysiological tests, such as nerve conduction studies (NCS), which evaluate the function of the peripheral nerves [102]. However, routine annual screening using clinical neurological examination is insensitive and often fails to detect early changes before irreversible damage [103]. The retinal vasculature, which reflects systemic microcirculation, may also play a role in the development of diabetic neuropathy. In a study, Benson et al. used a pre-trained VGG16 CNN and a SVM classifier to analyze retinal images and then compared AI-based retinal analysis with physician-diagnosed diabetic peripheral neuropathy. They attained 89% accuracy, 78% sensitivity, and 95% specificity for detecting diabetic peripheral neuropathy [104]. Among the DL methods evaluated for diagnosing diabetic neuropathy using CFP, SqueezeNet, Inception, and DenseNet emerged as the most effective. These models achieved AUCs of 0.8013 (± 0.0257) during validation and 0.7097 (± 0.0031) during testing, based on analyses performed on various datasets with and without pre-trained weights [105].

### Eyes on the kidneys: screening for chronic kidney disease

Diabetic nephropathy, a microvascular complication of DM, is a common cause of chronic kidney disease (CKD) [106–109]. The global prevalence of CKD is about 13.4% [110]. According to the Global Burden of Disease (GBD) study, CKD-related deaths increased by 41.5% between 1990 and 2017 [111]. In 2020, the World Health Organization ranked CKD as the tenth leading cause of death and projects that it will become the fifth leading cause by 2040 [112]. Early diagnosis by blood and urine screening assays, including urinary albumin-to-creatinine ratio (ACR), is recommended to identify at-risk patients and initiate interventions that slow the progression of kidney dysfunction. However, persistent albuminuria, which means ACR > 30 mg/g, and eGFR < 60 ml/min/1.73 m^2^, in the context of DM and in conjunction with other complications such as DR, are sufficient to establish the diagnosis of diabetic nephropathy [113, 114]. Retinal images provide clues to systemic microcirculatory health and may be used to screen for diabetic nephropathy. In a study by Sabanayagam et al. [115], clinical parameters and retinal images were utilized both independently and in combination within a CondenseNet DL model to predict diabetic nephropathy. The model achieved AUC values of 0.916 for clinical risk factors alone, 0.911 for retinal images alone, and 0.938 when both input types were combined. In a similar study, a ResNet50 DL model attained maximum AUCs of 0.861, 0.918, and 0.930 for clinical risk factors, retinal images, and hybrid inputs, respectively [116]. These findings corroborate the superior performance of AI-based diabetic nephropathy detection using combined clinical and retinal image inputs [117].

### Retinal images for diagnosing peripheral arterial disease

**Fig. 3:**
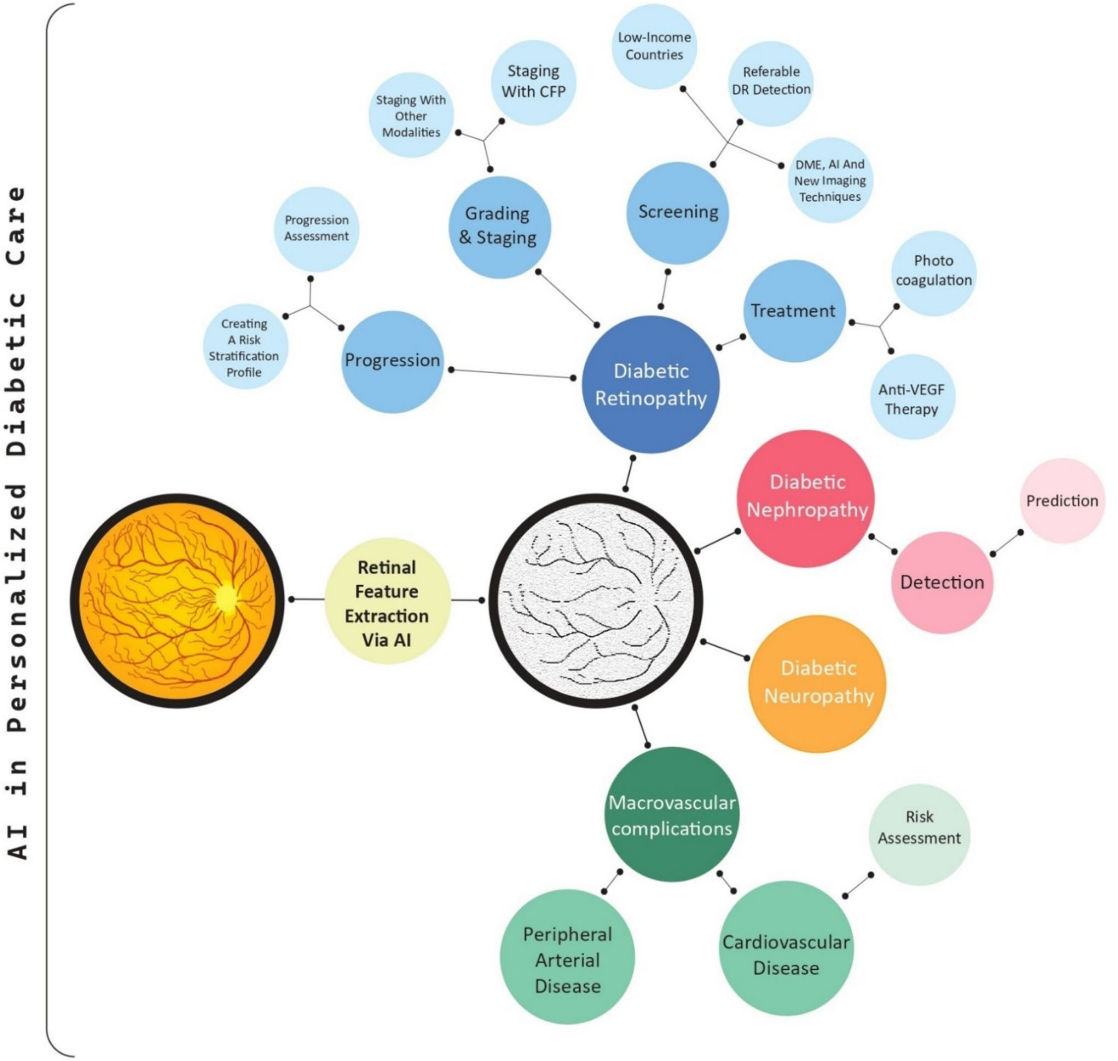
The diagram provides a structured overview of the integration of AI in diabetic care, showing the pathways in which AI has significant applications, including DR, nephropathy, neuropathy, cerebrovascular disease, peripheral arterial disease, and cardiovascular assessment. Each branch highlights the potential of AI to enhance diagnosis, monitoring, and management within specialized areas of diabetic care

Peripheral arterial disease (PAD) is a macrovascular complication of DM, with an incidence rate of 25–30% [118]. The elevated risk of PAD in type 2 DM (T2DM) can be attributed to a combination of traditional cardiovascular risk factors―age, sex, race, smoking, pulse pressure, glycated hemoglobin, albuminuria, and hyperlipidemia [119]―and diabetes-specific factors, including postprandial hyperglycemia, advanced glycation end-products, lipoproteins, and hypercoagulability [120]. A low ankle-brachial pressure index is commonly used to screen for lower limb PAD non-invasively, but may be falsely high in elderly people with inelastic arteries. Alternative noninvasive diagnostic techniques are ultrasound Doppler waveform analysis and toe-brachial index [121]. However, the gold standard technique for diagnosing PAD is Computed tomography angiography (CTA) [122]. Deep neural network architecture has recently been demonstrated to be a promising method for detecting PAD using CFP. The most successful model in this inventive methodology achieved an AUROC maximum score of 0.890. Moreover, visualizing the attention weights used by the network, provides valuable insights into its decision-making process, particularly the significance of ocular features in PAD. Statistical analysis of the model’s performance confirmed that the optic disc and temporal arcades were assigned significantly higher importance (*p* < 0.001) than the retinal background in the detection process. These results robustly support the feasibility and effectiveness of utilizing modern DL methodologies to detect PAD [123]. However, a major challenge in employing AI to diagnose PAD from retinal images is the difficulty of integrating high-quality images and assembling comprehensive training datasets with labeled PAD cases into the AI frameworks [124, 125]. Figure 3 shows a systematic overview of the use of AI in diabetes care.

**Table 1:** Summary of performance metrics of models and architectures for diabetes complications diagnosis

Study	AI modeling algorithms	Architecture	clinical predictors	Metric	Results
Diabetic Cardiovascular disease					
Poplin et al. [97]	DL	Inception-v3 (in the UKBiobank dataset)	Age	R2 (95% CI)	0.74 (0.73,0.75)
			Gender	AUC (95% CI)	0.97 (0.966,0.971)
			Smoking status	AUC (95% CI)	0.71 (0.70,0.73)
			BMI	R2 (95% CI)	0.13 (0.11,0.14)
			SBP	R2 (95% CI)	0.36 (0.35,0.37)
			DBP	R2 (95% CI)	0.32 (0.30,0.33)
			HbA1c	R2 (95% CI)	0.09 (0.03,0.16)
Diabetic Neuropathy					
Benson et al. [104]	DL, in conjunction with TL	VGG16 CNN	retinal changes as a predictor of diabetic neuropathy	Accuracy	89%
				Sensitivity	78%
				specificity	95%
Cervera et al. [105]	DL	SqueezeNet, Inception, and DenseNet	retinal changes as a predictor of diabetic neuropathy	AUC (95% CI)	0.8013 (± 0.0257)
Diabetic Nephropathy					
Sabanayagam et al. [115]	DL	CondenseNet	Image only showing retinal changes as a predictor of diabetic nephropathy	AUC (95% CI)	0·911 (0·886–0·936)
				Sensitivity	0·83
				Specificity	0·83
			RF only includes age, sex, ethnicity, diabetes, and hypertension	AUC (95% CI)	0·916 (0·891–0·941)
				Sensitivity	0·82
				Specificity	0·84
			hybrid DLA combining image and RF	AUC (95% CI)	0·938 (0·917–0·959)
				Sensitivity	0·84
				Specificity	0·85
Zhang et al. [116]	DL (CNN)	ResNet-50	Image only showing retinal changes as a predictor of diabetic nephropathy	AUC	0.829–0.918
			RF only (age, sex, height, weight, body-mass index, and blood pressure)	AUC	0.787–0.861
			hybrid DLA combining image and RF	AUC	0.845–0.930
Diabetic peripheral arterial disease					
Mueller et al. [123]	DL (CNN)	Multiple Instance Learning (MIL)	retinal changes as a predictor of diabetic PAD	Accuracy	0.674–0.837
				AUROC	0.653–0.890

## AI techniques used in retinal images analysis

**Fig. 4:**
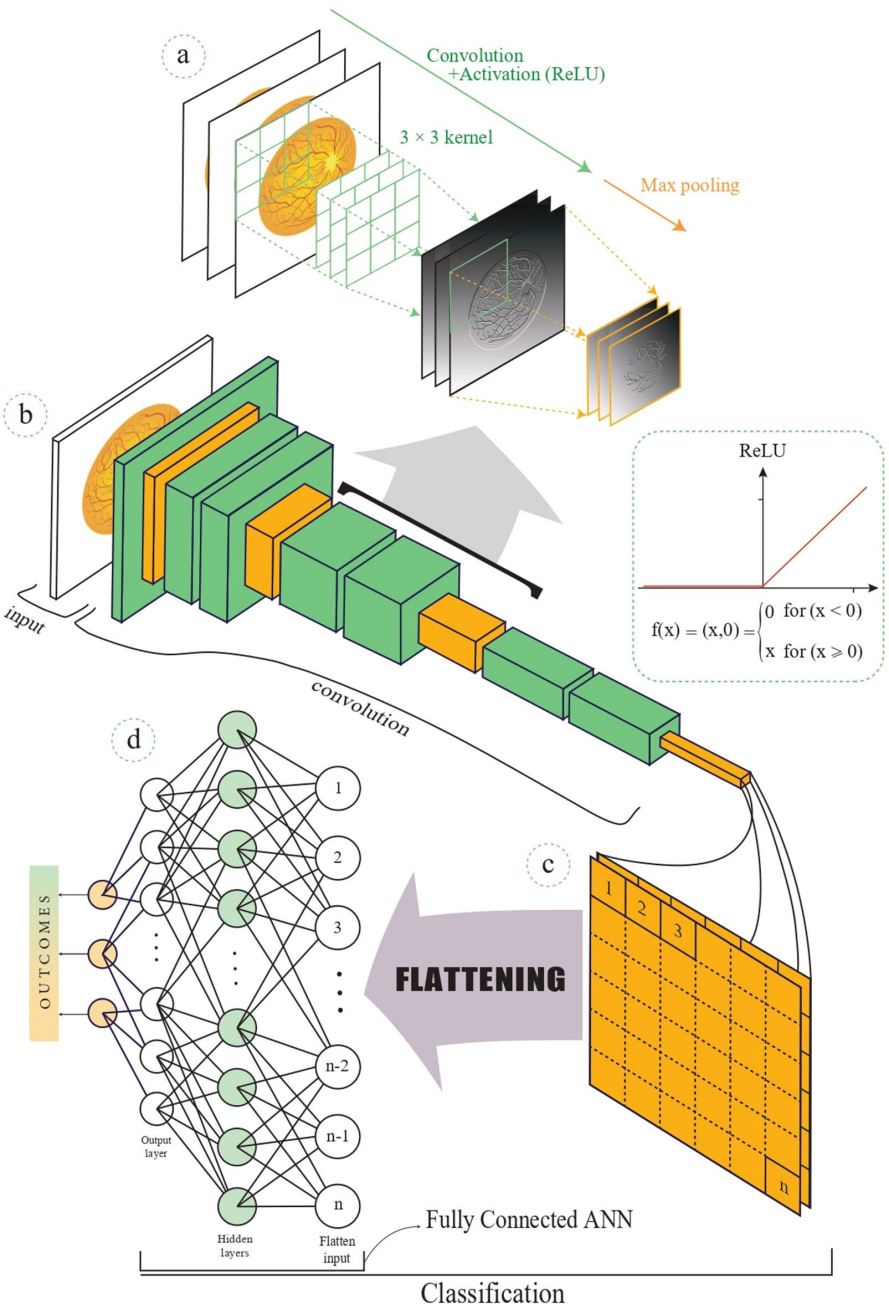
A detailed look at the architecture of a convolutional neural network (CNN) for image analysis. a: The convolutional layers (green) use kernels that slide over the three-channel RGB image to recognize key features from the input image. Following the convolution process, the Rectified Linear Unit (ReLU) activation function (green) is applied to introduce non-linearity, enhancing the network’s capability to learn intricate patterns. Subsequently, the max pooling process (orange) is applied, reducing the spatial dimensions by selecting the maximum value within specified regions. **b**: this part is the multiple repetitions of convolution, ReLU activation function, and max pooling processes, creating the final feature map. **c**: shows flattening of the last max pooling layer, which converts the 2D feature maps into a 1D vector to prepare the data for the upcoming fully connected layers. **d**: the architecture transitions to fully connected layers, leading to a classification process where the features are used to provide definitive conclusions

AI researchers have used various models to analyze retinal images; the two most common neural networks are the CNN-based architectures ResNet [125–127] and VGGNet [128, 129]. Figure 4 illustrates the architecture of a CNN specifically designed for image analysis. Deep neural networks often fail in practice due to difficulties in optimizing the networks caused by the diminishing gradient problem. ResNet overcomes this issue by residual learning, which uses skip or shortcut connections to skip one or more layers during forward and backward passes [125, 130]. This ensures that the deeper layers produce no higher training errors than their shallower counterparts, which makes it easier for the network to learn identity functions. ResNet’s modular architecture also facilitates up or down scaling of the model by adjusting the number of residual blocks. This makes it an ideal choice for developers because they can tailor the architecture to specific needs and computational constraints. Belying its depth, ResNet consumes comparatively fewer computational resources, which underscores its efficiency. VGGNet is a family of deep CNNs distinguished by its simple design, ability to capture complex image characteristics, and excellent image classification performance. VGG16, a notable variant comprising 16 layers [128, 129], begins with layers containing 64 channels, with a 3 × 3 filter size and consistent padding, which are succeeded by a max-pooling layer with a stride of (2, 2), and subsequent convolution layers that progressively increase in channels, to for example 128, while maintaining a uniform 3 × 3 filter size. VGGNet is predominantly engineered for image classification on extensive datasets, notably the ImageNet database, which encompasses over 14 million images categorized based on the WordNet structure. Beyond image classification, advancements in VGG architectures have been pursued to cater to diverse computer vision applications and to augment its efficacy in classification problems [131, 132].

Inception-v3, DenseNet, AlexNet, ResNet, and U-Net are neural network architectures with distinct structural variations that have been applied to retinal image analysis [133]. Inception-v3, from the GoogLeNet family, was designed for multi-level feature extraction tailored to large-scale image recognition tasks, incorporating factorization and other approaches, such as batch normalization, to minimize parameters and maximize performance [134, 135]. DenseNet is well-suited for image classification and segmentation due to dense connections between layers, which improve gradient flow and encourage feature reuse [136]. AlexNet, an early CNN, is adept at image classification due to its deep architecture and use of the rectified linear unit (RELU) activation but may be outperformed by later models in terms of efficiency [137]. By expanding on residuals and using split-attention methods, ResNet is able to train the model to focus on the most relevant characteristics for image classification [138]. Unlike Inception-v3 and AlexNet, which are more general-purpose models, U-Net was developed specifically for segmenting biological images [139]. Its “U-shaped” design allows for more accurate localization, which is important in diagnostic imaging [140].

Supervised ML learns by mapping the input signals or images to their respective labels. Supervised ML techniques have been extensively employed in retinal image analysis for the detection and classification of various retinal diseases, including DR. With labeled retinal images, the algorithms can effectively learn the patterns associated with DR, thereby facilitating early diagnosis and timely interventions to avert irreversible vision loss [25, 141]. TL uses pretrained datasets, such as ImageNet [142], to overcome the need for large retina-specific training datasets [143]. For retinal image analysis, the final layers of the pre-trained model are typically adjusted to cater to the specificities of the retinal diseases or features. After this adjustment, the model is fine-tuned on available retinal datasets. TL accelerates the training process, often yielding models with enhanced accuracy and robustness that can be applied to diverse tasks including DR detection, retinal lesion identification, and retinal vessel segmentation [25, 62]. A recent study analyzing the morphological characteristics of retinal vessels in DR patients demonstrated that TL can accurately quantify vascular changes, revealing significant differences between DR patients and healthy individuals, and showing that DME does not alter the overall retinal vascular pattern [144].

**Fig. 5:**
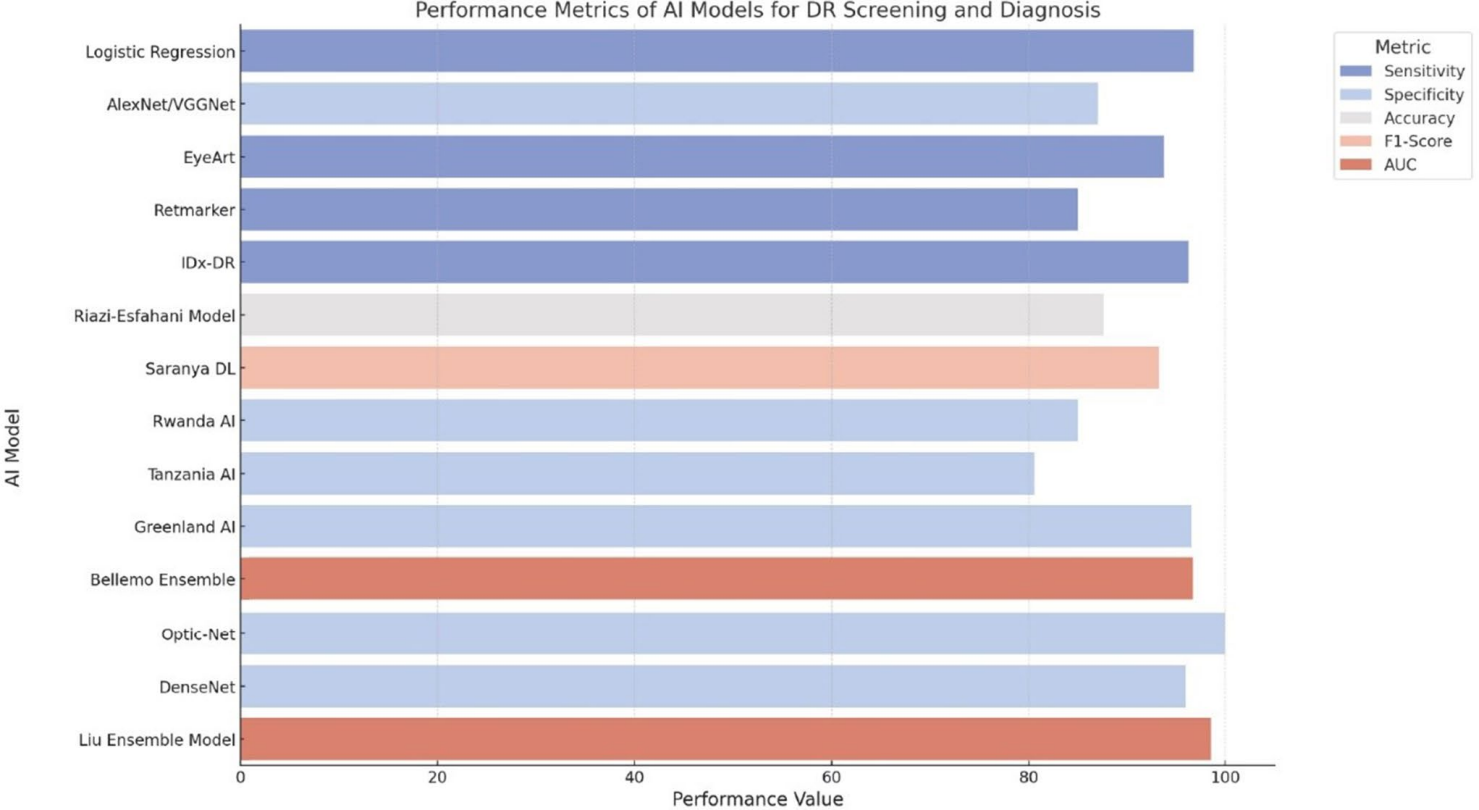
Performance comparison of AI models across the best metrics for DR screening and diagnosis, as obtained from the included studies. All AUC values are multiplied by 100

Various AI models are compared in terms of sensitivity, specificity, F1-score, and AUC for diagnosing and screening DR complications, as shown in Fig. 5. This highlights the quality and weakness of each model. For example, Logistic Regression and Saranya DL achieved high sensitivity (more than 96%), whereas the EyeArt and Bellemo Ensemble models did not exceed specificity and showed a balanced result between sensitivity and specificity. Optic-Net is a model that has maximum specificity (100%) and high accuracy (98%), making it the most dependable for DR screening with minimum false positives. Conversely, specificity, one of the important characteristics of the Rwanda AI model, is only moderate, which means there is a wide scope for further improvement, specifically when resources are limited.

Such diversity in the performance of AI represents AI’s ability to fit in with a range of clinical needs. For instance, if the sensitivity of a test is extremely high, then many cases can be detected. Conversely, if the specificity is the main goal of a test, not so many referrals will be made. These indicators show that Optic-Net was the best model in terms of precision, whereas Saranya DL and Bellemo Ensemble adhered to the criteria of sensitivity and specificity, thus allowing broader screening. The Liu Ensemble Model also performs well, with an impressive AUC of 0.981, showing strong overall diagnostic reliability. The best choice depends on the clinical priorities and whether precision or balance is more critical.

**Fig. 6:**
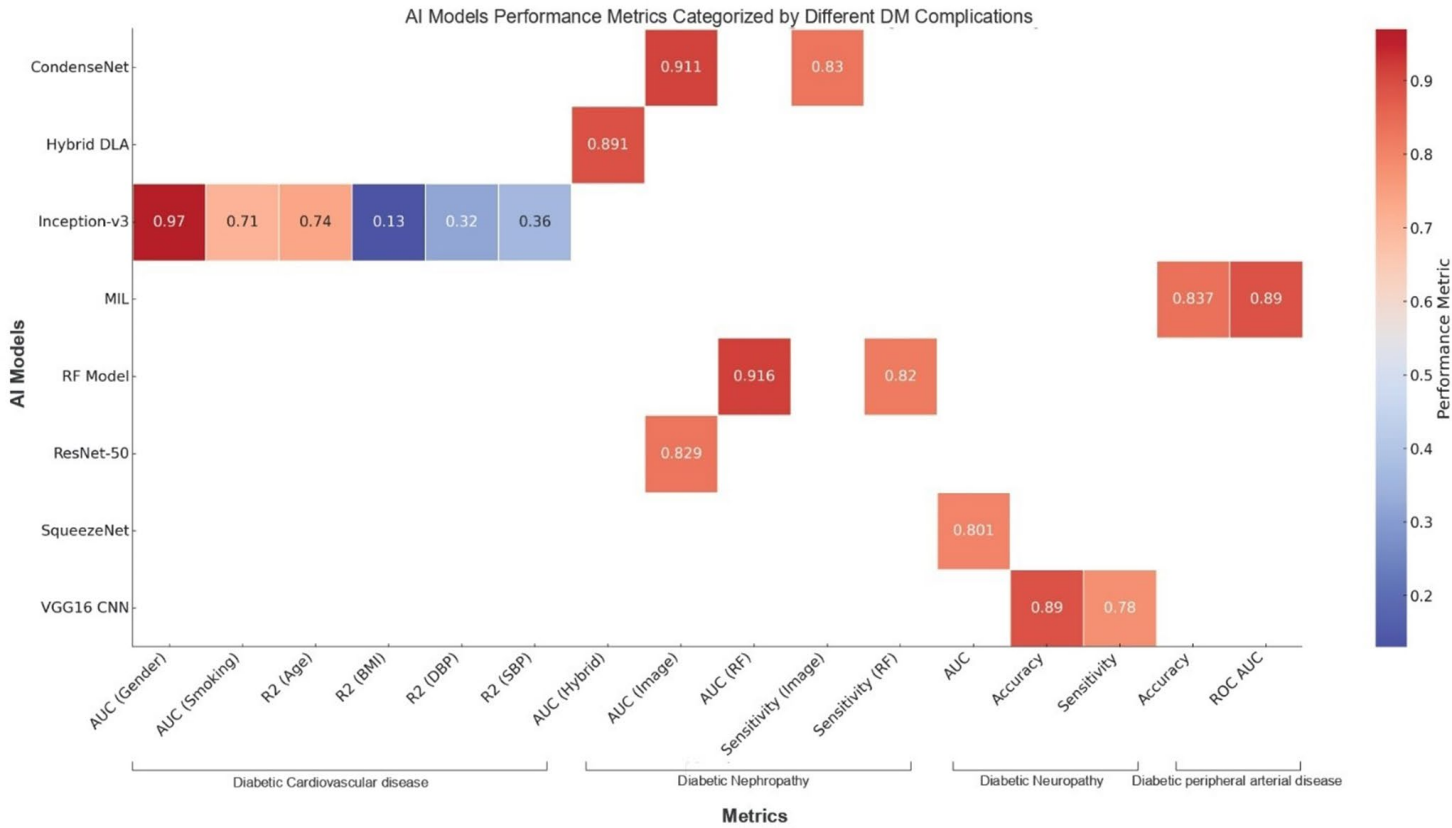
Comparison of AI model performance across various metrics for diabetic complication diagnosis, based on data extracted from the included studies

The nuanced interplay between the AI models and their performance metrics in diagnosing diabetic complications is shown in Fig. 6. Models such as VGG16 CNN exhibit strong specificity (0.95), making them reliable for reducing false positives in clinical screening. High AUC values, as seen with Hybrid DLA and Inception-v3, reflect their robust discriminatory power for differentiating cases, ensuring accurate diagnostic outcomes. Sensitivity-focused models, such as CondenseNet, effectively minimize missed diagnoses, which are critical for early detection in high-risk populations. Accuracy, exemplified by MIL and VGG16 CNN, highlights a model’s overall reliability across diverse settings. This heatmap also reveals key patterns, such as the consistent link between models excelling in sensitivity and those designed for nuanced clinical predictions and the role of metrics such as AUC in indicating broad diagnostic capabilities. However, models optimized for specificity, like RF, may exhibit slightly lower sensitivity, suggesting trade-offs between reducing false positives and ensuring inclusivity in detection. While these performance metrics provide a holistic view of AI model effectiveness, contextual factors like patient demographics, imaging protocols, and dataset variability must be considered to ensure the findings’ clinical applicability and reliability.

## Challenges of AI in diabetes care

Only two Asian countries have DR screening programs that conform to International Council of Ophthalmology standards [145]. There is not only an imperative to standardize national protocols to mitigate gaps in screening and referral timelines [146] but also a need to establish comprehensive guidelines for AI implementation in DR screening to ensure standardized and effective practice [147]. AI-based DR screening can reduce economic constraints and enhance accessibility to healthcare services [148], but several obstacles remain to surmount [149]. Overcoming these challenges will require multidisciplinary cooperation, data standardization, resource sharing, real-world verification, and productization [150]. In particular, DL algorithms require large datasets with thousands or millions of images for training, which are costly to label and curate.

AI developers often resort to using available but limited training datasets, which may not be generalizable to the real world, where image quality may be affected by deficiencies in the clinical setting and are not of consistently high quality [151]. Suboptimal image quality and low target pathology incidence may drive higher false-positive rates [152]. Even if image issues are resolved, AI algorithms that only focus on retinal image assessment will not be able to analyze the clinical and psychosocial aspects that can modulate the diagnosis, perception, coping mechanisms, and holistic management of individual patients [153].

The lack of compatibility among electronic health record vendors impedes such clinical data integration and may delay the execution of computer-interpretable guidelines for clinical decision-making in DR [154]. To garner wider adoption, there is a need for more research on the assessment and testing of AI diagnostic tools in clinical settings [155], even as validating AI algorithms in different populations and camera systems remains challenging [156]. Additionally, conducting rigorous clinical trials of AI models, particularly randomized controlled trials, requires meticulous methodological adjustments and considerations of clinical equipoise, informed consent, and fairness [157]. Practical challenges to building and deploying AI at scale include regulatory pressures, conflicting business goals, and data quality issues [158]. Even as AI promises solutions to problems through technological advances, AI in healthcare raises ethical and legal issues [159]. As AI systems become more autonomous, the need to incorporate ethical considerations and moral reasoning into model decision-making processes has become more pressing [160]. Equally important is the obligation to identify and resolve ethical issues―inclusivity, bias, and social acceptability―to ensure fair user access to promising healthcare AI technologies [161]. Issues such as protecting sensitive medical information during image analysis or remote sharing via tele-retinal screening [162] also raise patient privacy concerns, requiring careful system security during AI solution development and deployment.

Current approaches to AI model design are delinked mainly from the complex healthcare environments they are intended for; as a result, the development of AI models has vastly outpaced their adoption into existing clinical workflows [163]. This separation contributes to models that lack clear use cases and are neither tested nor scaled in clinical settings. A mixed-methods approach that integrates design thinking and quality improvement methodologies―aiming to understand variations in healthcare processes and incorporating user-centered design to ensure model functionality in practice [164]―can potentially compensate this gap to smoothen AI integration within the healthcare domain [165], and gather wider clinical adoption [166]. Moreover, ensuring that AI models are interpretable is crucial for building trust among clinicians [167]. Techniques like Gradient-weighted Class Activation Mapping (Grad-CAM) play pivotal roles in this regard by generating visual explanations that highlight regions in retinal images significantly influencing the model’s decisions, thereby allowing clinicians to verify that AI systems focus on medically relevant areas [168]; moreover, integrating Grad-CAM with local interpretable model-agnostic explanations (LIME) further enhances explainability by enabling visualization of the decision-making process, which in turn improves trust in AI-generated outcomes [169]. For instance, Mercaldo et al. utilized Grad-CAM with CNN to differentiate between healthy eyes and those with DR, achieving an accuracy of 98%, and further distinguishing between stages of DR with 91% accuracy. Their study also introduced a similarity index to evaluate the robustness of heatmaps generated by class activation mapping algorithms, ensuring consistent localization of symptomatic areas in angiography [170].

Real-time AI processing can analyze retinal images within seconds, facilitating quicker diagnoses and timely interventions, which is crucial for preventing disease progression in diabetic patients [171]. Ruamviboonsuk et al. conducted a prospective study in Thailand to evaluate a DL system for real-time DR detection in a community-based screening program. The system demonstrated 94.7% accuracy, 91.4% sensitivity, and 95.4% specificity in detecting vision-threatening DR, comparable to retina specialists. This study emphasizes the importance of integrating socioenvironmental factors and workflows into large-scale AI-driven screening programs in low- and middle-income countries [44]. Natarajan et al. explored the use of an real-time AI system, Medios AI, for smartphone-based retinal imaging by minimally trained health workers in Mumbai, India. The system achieved a sensitivity of 100% and specificity of 88.4% for referable DR detection. These results highlight the potential of AI systems in community-level DR screening, particularly in remote areas lacking access to ophthalmologists [171].

AI screening systems have shown promising results in detecting DR from CFP and OCT images. Despite the advancements, there remains a critical need for validation, regulatory frameworks, safe implementation, and demonstration of clinical impact before these innovations can be widely adopted on a large scale. Testing AI models in clinical settings is crucial for identifying and addressing system issues before full deployment [172, 173]. Additionaly, AI tools must be validated and calibrated for local populations and clinical contexts, as results from one setting may not be universally applicable [174]. Burlina et al. [175] demonstrated that AI tools trained on data from specific population groups can exhibit disparities in performance when applied to other groups, which introduce biases in the diagnosis of DR. Similarly, Rogers et al. [176] found that AI models trained on fundus images captured using standard desktop fundus cameras failed to provide accurate diagnoses when evaluated on images taken with handheld portable cameras. This highlights how variations in imaging device configurations can negatively impact model performance. These findings showes the importance of training AI models on diverse datasets that encompass various demographics and imaging conditions to enhance their generalizability and ensure equitable healthcare outcomes. To further address these challenges, tailored solutions, such as localized AI systems designed for specific regions or populations and collaborative initiatives sensitive to cultural and socioeconomic contexts, could significantly improve model performance across varied patient groups [177].

Interpretability is essential for gaining the trust of clinicians and patients alike, in complex AI models of DR diagnosis or treatment decision-making. There is a need for transparent and interpretable AI in the reasoning processes behind the generated model outputs. Explainable AI (XAI) refers to systems that provide understandable explanations for their decisions and actions. While explainable AI is an active area of research, achieving reliable and interpretable explanations remains a significant challenge [178].

Finaly, regulatory compliance is also a crucial function that allows for the application and operation of AI models in the context of DR diagnosis while also ensuring their safety, efficiency, and universality. After passing through the demanding FDA process, which involves extreme validation, the like of IDx-DR can demonstrate, the highest sensitivity and specificity in clinical settings. This example serves to highlight the transparency of the algorithm development process, the careful reporting of validation results, and the continuous monitoring of the post-market period [15]. Aligning with regulatory standards by promoting collaborations within the international setting creates AI research space from the challenges it faces and thus allows for the safe practice of AI in clinical care [177].

## Potential enhancements and future directions of AI in DR management

At the intersection of telemedicine and AI, tele-retinal image analysis promises to democratize access to screening and downstream healthcare services, transform the management of DR, and improve long-term patient outcomes while reducing financial and time costs for both patients and payers. AI-based DR screening has demonstrated encouraging outcomes, with DL algorithms yielding high levels of sensitivity and specificity. The operational efficiency of community-based tele-retinal image analysis may be enhanced: the gradability of retinal images can be assessed at source, expediting identification of poor-quality images for either manual or additional AI-based grading (the ultimate choice will depend on the associated labor cost vs. intrinsic diagnostic value-add of AI) [179–181]. Future technological developments in AI can introduce significant opportunities for technical refinements to optimize DR diagnosis and downstream management. Faster AI software processing speeds will enhance system accuracy and efficiency, facilitate seamless and responsive navigation of the AI interface by clinician users, and enable more effective preventive therapeutic interventions. Independent of these developments, real-world implementation of AI technology presents its practical challenges, e.g., workflow integration, technical adaptability, ethical implications, cost-effectiveness considerations, etc. To ensure the ethical and balanced integration of AI into DR screening programs, the governance model for AI implementation must focus on honesty, equality, reliability, and responsibility [172–174].

Ensemble learning is a machine learning approach that does accurate diagnostics in medical applications by using multiple algorithms to make predictions that are more reliable and robust. Methods like bagging, boosting, and stacking are usually employed in this method. Bagging distributes data through different subsets and trains multiple models. Boosting which is the sequential improvement of the previous models, was demonstrated in modeling liver and diabetes. Stacking, which involves using another model (called a meta-learner) to combine predictions from the multiple models, has been proven to be the most effective among other methods in various diseases [182]. However, they do not only decrease the risk of overfitting but also increase a model’s capability of generalizing over different datasets which is one of the reasons they are widely used in clinical settings. Moreover, ensemble learning is good for coping with problem areas like imbalanced datasets, which are usually seen in medical diagnostics, by strengthening model stability and reducing false outcomes [183]. Ensemble learning techniques, which combine multiple models, have shown to significantly improve accuracy in DR detection and classification compared to single-model approaches. Mondal et al. proposed an automated ensemble of DenseNet101 and ResNeXt models, combining DenseNet’s efficient feature utilization with ResNeXt’s advanced split-transform-merge strategy. Their approach, applied to preprocessed datasets (APTOS19 and DIARETDB1), achieved high accuracy for both two-class (96.98%) and five-class (86.08%) classifications, with robust precision and recall, particularly aided by GAN-based data augmentation to address class imbalance [184]. Similarly, Lukashevich et al. explored ensemble learning with a focus on hyperparameter optimization, combining grid search and random search techniques. Their gradient boosting model achieved strong performance, with a binary classification accuracy of 94% and a multi-class staging accuracy of 75.31%. Both studies highlight ensemble learning’s capacity to improve DR screening and staging, emphasizing the importance of integrating advanced algorithms and robust data preprocessing to achieve clinically relevant outcomes [185].

Explainable AI make the complex decision-making processes of AI models transparent and interpretable, which is essential, given the clinical impact of healthcare decisions, for gaining the trust and acceptance of AI model outputs by doctors and patients. The applications of explainable AI in the medical domain are vast and transformative [186, 187], encompassing diverse tasks, such as decision-making, risk management, predictions, and medical image analysis (the sensitivity of AI for detecting abnormalities often surpasses that of the human eye). Explainable AI aids in explaining AI-driven insights. For example, while a traditional AI model may provide a diagnosis with a confidence score (“DR detected with 95% confidence”), ophthalmologists may hesitate to Google Net trust the result or administer recommended treatment without knowing the basis for the diagnosis. With explainable AI, the system can highlight regions in the retina or specific lesions, like hemorrhages or microaneurysms, critical to the model decision via heat maps. These allow clinicians to relate to the results like traditional funduscopic examination, and the maps may additionally serve as useful guides to the planning of photocoagulation therapy, where applicable [188, 189].

**Fig. 7:**
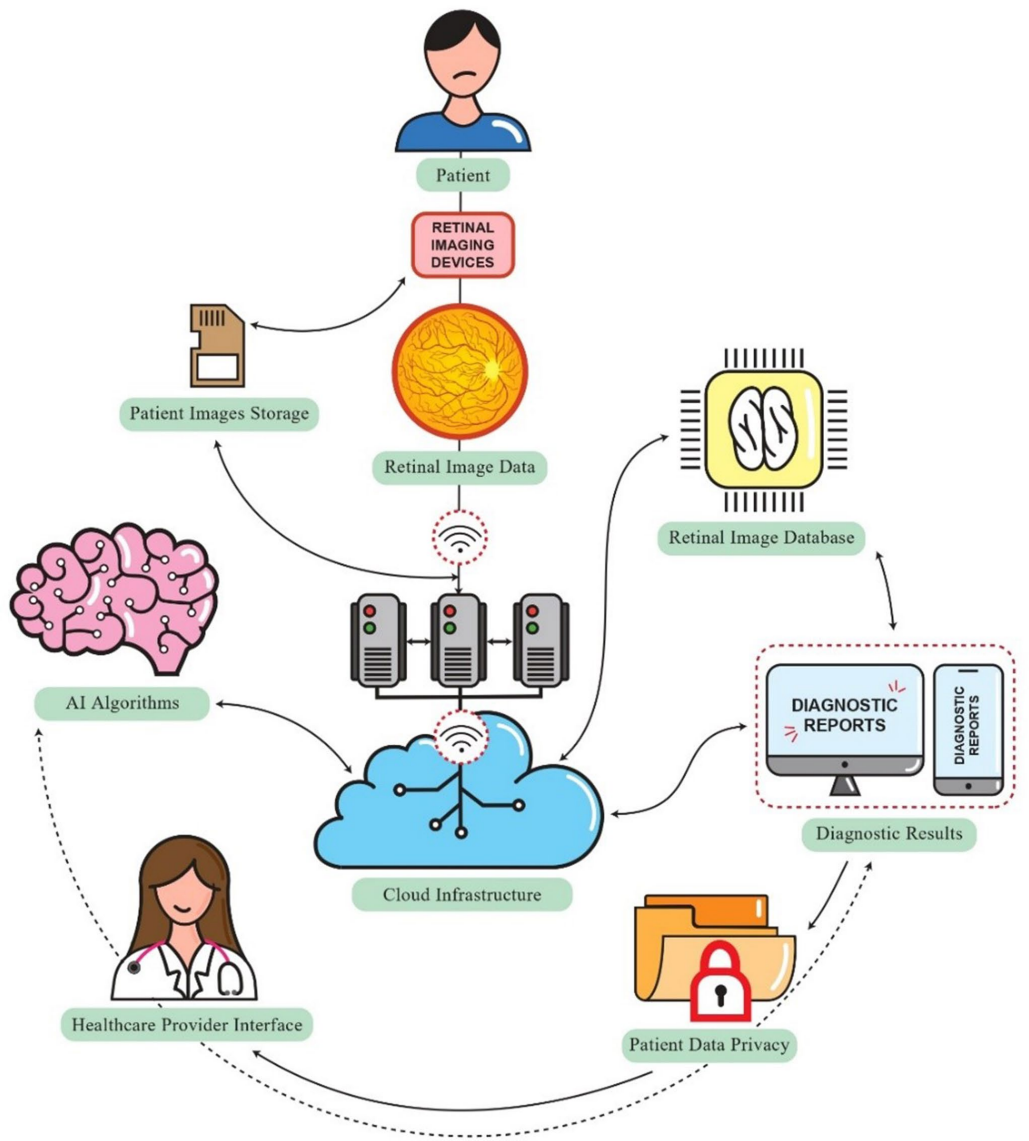
This schematic illustrates the interconnected components of a cloud-based system for DR screening. The cloud infrastructure, depicted as remote servers and databases, processes retinal image data captured by a retinal imaging device, such as a fundus camera or OCT scanner. AI algorithms analyze the images within the cloud, generating diagnostic results like risk scores for DR. These results are accessible to healthcare providers through interfaces on computers or mobile devices, ensuring prompt patient care. Patient data privacy measures safeguard sensitive information, including encryption and secure transmission protocols. Additionally, a feedback loop may exist, where diagnostic results contribute to the continuous improvement of AI algorithms over time

Cloud-based systems revolutionize diabetes management and prevention by enhancing data processing, enabling real-time interventions, and optimizing resources. They facilitate early detection, personalized therapy, and swift glucose fluctuation response. Migration to cloud structures reduces costs and administrative burdens, while user-friendly digital platforms support self-monitoring and community engagement. Integrating AI with cloud platforms promises sharper insights for combating diseases like Type II diabetes, leading to societal benefits. Moreover, some researchers have investigated the potential of cloud-based systems for diabetes management and prevention [190, 191]. A study by Salari et al. [192] has shown promising results in using mobile and cloud systems to improve self-care for chronic conditions, offering hope for more effective and user-friendly solutions. Similarly, Nasser et al. [193] have proposed innovative methods using advanced technology, such as AI and cloud computing, to predict glucose levels and integrate them with wearable devices. These advancements point toward a future where cloud-based systems could revolutionize how diabetes is managed, offering personalized and timely interventions to enhance health outcomes for individuals with the condition. Figure 7 illustrates the interconnected components of a cloud-based system for DR screening.

## Conclusion

Given the increasing prevalence and incidence of DM, developing cost-effective, population-based DR screening and management strategies is becoming increasingly important nationally. AI has shown significant promise in accurately diagnosing DR, with potential applicability extending beyond the diagnosis and grading of DR. This includes the diagnosis of diabetic neuropathy, diabetic nephropathy, and cardiovascular diseases. These applications may involve using CFP independently or in conjunction with other advanced diagnostic techniques, such as OCT and various clinical parameters. AI’s capacity to assess a patient’s health about DM complications and to forecast the risk of future complications positions AI-assisted retinal image analysis as a potentially key component in personalized medicine for individuals with DM.

## Data Availability

N/A.
